# Providers' knowledge of diagnosis and treatment of tuberculosis using vignettes: evidence from rural Bihar, India

**DOI:** 10.1136/bmjgh-2016-000155

**Published:** 2016-12-16

**Authors:** Manoj Mohanan, Jeremy D Goldhaber-Fiebert, Soledad Giardili, Marcos Vera-Hernández

**Affiliations:** 1 Sanford School of Public Policy, Duke Global Health Institute, and Department of Economics, Duke University, Durham, North Carolina, USA; 2 Centers for Health Policy and Primary Care and Outcomes Research, Stanford University School of Medicine, Stanford, California, USA; 3 School of Economics and Finance, Queen Mary University of London, London, UK; 4 Department of Economics, University College London, London, UK

## Abstract

**Background:**

Almost 25% of all new cases of tuberculosis (TB) worldwide are in India, where drug resistance and low quality of care remain key challenges.

**Methods:**

We conducted an observational, cross-sectional study of healthcare providers' knowledge of diagnosis and treatment of TB in rural Bihar, India, from June to September 2012. Using data from vignette-based interviews with 395 most commonly visited healthcare providers in study areas, we scored providers' knowledge and used multivariable regression models to examine their relationship to providers' characteristics.

**Findings:**

80% of 395 providers had no formal medical qualifications. Overall, providers demonstrated low levels of knowledge: 64.9% (95% CI 59.8% to 69.8%) diagnosed correctly, and 21.7% (CI 16.8% to 27.1%) recommended correct treatment. Providers seldom asked diagnostic questions such as fever (31.4%, CI 26.8% to 36.2%) and bloody sputum (11.1%, CI 8.2% to 14.7%), or results from sputum microscopy (20.0%, CI: 16.2% to 24.3%). After controlling for whether providers treat TB, MBBS providers were not significantly different, from unqualified providers or those with alternative medical qualifications, on knowledge score or offering correct treatment. MBBS providers were, however, more likely to recommend referrals relative to complementary medicine and unqualified providers (23.2 and 37.7 percentage points, respectively).

**Interpretation:**

Healthcare providers in rural areas in Bihar, India, have low levels of knowledge regarding TB diagnosis and treatment. Our findings highlight the need for policies to improve training, incentives, task shifting and regulation to improve knowledge and performance of existing providers. Further, more research is needed on the incentives providers face and the role of information on quality to help patients select providers who offer higher quality care.

**Key questions**

**What is already known about this topic?**

- Tuberculosis (TB) drug resistance is a major public health challenge globally and in India; a key contributing factor is poor knowledge of TB diagnosis and treatment among providers.
- Evidence from systematic reviews shows long diagnostic delays for TB, with patients frequently switching providers—suggesting they do not receive appropriate care that they are satisfied with.
- Recent studies in India have also demonstrated gaps between what providers know and what they do in clinical practice—the know-do gap.

**What are the new findings?**

- This study measures providers' knowledge of diagnosis and treatment of TB, using a sampling method designed to estimate competence of providers who are most commonly visited by households in study areas.
- In addition to finding low levels of provider competence in diagnosis and treatment of TB, we also find evidence suggesting that, among all providers who report that they treat TB cases, there is no significant association between having formal medical training and provider competence. Providers with medical training were not more likely to diagnose or treat TB correctly compared with those without formal training.
- Our analysis also demonstrates the severe information asymmetry problems in the healthcare market because of which patients in these settings are unable to rely on common signals of quality such as medical degrees or experience to infer provider competence.

**Recommendations for policy**

- Our results highlight the need for policies to improve training, incentives, task shifting and regulation to improve knowledge and performance of existing providers in the healthcare system.

## Introduction

The scale of India's tuberculosis (TB) burden looms large, contributing almost a quarter of the 9.6 million cases worldwide.^1^ Bihar, one of India's largest and poorest states, bears a substantial share of India's TB burden, as active TB disease is associated with poverty.^2–4^ With over 100 million inhabitants and a per-capita annual income of $502^i^ in 2011, less than half of India's national average, Bihar's healthcare system registered almost 68 000 new TB patients in 2015 alone.^5^ ^6^

A major challenge to achieving improved outcomes in Bihar is the generally poor quality of medical care available to patients in rural areas.^7^ In the public sector, lack of availability of trained providers and absenteeism among medical care providers is a key limitation—as high as 67% in primary health centres among doctors and 52% among nurses.^8^ ^9^ Not surprisingly then, over 90% of the healthcare used by households (outpatient care) in rural Bihar is provided by the private sector, of which 70% is from informal sector providers.^10^ The net result of widespread absenteeism in the formal public sector, untrained informal sector providers and low levels of provider effort is the alarmingly low quality of care provided to patients.

Active TB disease is treatable, and multidrug resistance potentially avoidable, provided that cases can be correctly diagnosed and appropriate treatment regimens are administered for adequate periods of time. However, systematic reviews from India have shown long diagnostic delays with patients frequently switching providers,^11^ and have also shown poor quality of TB care in India.^12^ Previous studies of rural patients in Bihar treated for TB using DOTS (Directly Observed Treatment, Short course) in the public sector show high rates of drop-out and symptom persistence, despite completing treatment.^13^ Another study that sampled 371 174 individuals in 30 districts across India shows that nearly half of those with TB who sought care did so in private sector and non-DOTS settings.^14^ Yet, the potential for higher quality TB diagnosis and treatment as represented by the limits of provider knowledge remains ill characterised in settings like Bihar, especially among private sector providers who are the first point of medical contact for most rural patients. There are no statewide efforts to engage the informal private sector in TB control.

This paper contributes to the literature on quality of care in rural areas in developing countries, focusing on the diagnosis and treatment of TB—a chronic, communicable disease of global significance.^1^ We estimate the knowledge of healthcare providers in rural Bihar, India, in terms of providing a correct diagnosis of TB and prescribing the appropriate treatment when interviewed using clinical vignettes. We also analyse how healthcare provider characteristics that are observable to patients predict the probability of a correct diagnosis and treatment for the vignettes. Findings from these analyses have important public health and policy implications for improving low levels of provider knowledge and increasing the quality of TB diagnosis and treatment.

## Methods

### Setting

We analyse data from provider quality assessments undertaken as part of baseline surveys conducted for the Bihar Evaluation of Social Franchising and Telemedicine (BEST) project—an evaluation of a large telemedicine programme funded by the Bill and Melinda Gates Foundation in Bihar.^15^

### Sampling method

The provider surveys and quality assessments were conducted in 80 randomly selected clusters (out of 360 rural clusters) in the study, representing rural areas from 11 districts. Clusters in the study were defined to represent market catchment areas with a population of ∼20 000 as described in Mohanan *et al*.^16^ The objective of the provider surveys was to assess the knowledge of the providers who deliver care to households surveyed at baseline. In each cluster, data from interviews with 64 randomly selected households were used to generate a list of all providers visited in the past 6 months, regardless of medical training of the providers. The number of households was chosen based on power calculations to estimate the impact of the programme on population health outcomes.^16^ We selected the five most frequently visited providers as reported by the 64 randomly selected households in each cluster for inclusion in the present study, in order to have 90% power to detect a 20% improvement in quality of care provided. The total number of providers in the study areas ranged between 6 and 70;^16^ the current study focuses on the 5 most commonly visited providers in these clusters. Since some clusters had fewer than 5 providers, our final sample includes 395 providers. We administered surveys as well as a series of clinical vignettes to each sampled provider, including a vignette for a case of suspected pulmonary TB. The BEST study protocol was approved by Duke University (29755) and India's Health Ministry Steering Committee (number 12/2008/30-HMSC/4).

### Data collection instruments

We use data from the provider surveys and provider responses to vignettes to assess the quality of care available for TB patients. Trained interviewers first administered a detailed structured survey to each sampled provider. The survey collected information on provider characteristics, such as age, education, medical qualifications, experience, types of clinical activities in practice, types of illnesses treated and infrastructure in the facility in which they practice.

To measure provider knowledge, we analyse data from clinical vignettes. The vignette method involves presenting a hypothetical case to the provider in an interview setting with two interviewers, one reading out scripted answers to the provider's questions and the other recording all of the provider's responses to the vignette. The case intends to represent a new pulmonary TB patient, who is visiting a healthcare provider for the first time, with productive cough of more than 2 weeks, accompanied by chest pain, haemoptysis, loss of appetite, weight loss, night sweats and fever.

The TB vignette starts with the interviewer telling the provider to assume that a man aged 40 years visits the provider and that he will comply with all tests and medications that the provider might recommend and will return if required. The patient reports, “Doctor, I have been suffering from fever, cough and weakness, and I have been losing weight.” The provider then proceeds to ask history questions (eg, ‘Do you have fever with chills?’) and the interviewer reads out the scripted responses (‘No’). If the provider says she would examine the patient and check his pulse, respiratory rate or auscultate his chest, the interviewer will read out the appropriate responses (‘80 bpm’, ‘20 breaths/minute’, ‘normal breath sounds’, respectively). Similarly, the vignettes also provide results on tests that the provider might recommend—if specific blood tests were recommended, the enumerators would read out test results. An X-ray was provided on request showing opacity in the right apex. After pilot testing, and based on previous studies conducted in a range of settings,^17^ as well as inputs from local clinicians, the vignettes were designed to include clinically relevant information as well as information that were commonly asked for by providers for social or cultural reasons (such as marital status or number of children). (See vignette modules included in online supplementary appendix).

The vignette responses provide information about whether the doctor is able to ask the most clinically relevant questions, to establish the correct diagnosis and also detailed information about the investigations recommended and treatment prescribed. Analyses of the providers' characteristics in relation to their performance on the vignettes (ie, correctly diagnosing and offering appropriate treatment) form the core of our analysis.

### Statistical analysis

Descriptive statistics for provider characteristics were computed to compare groups of providers with and without medical qualifications. Differences between these two groups were tested using unpaired two-tailed t-tests and χ^2^ tests of proportions, with SEs adjusted for the study design.

We assessed providers' performance on vignettes based on: (1) correct diagnosis; (2) correct treatment and (3) recommendation of a referral to another provider or hospital. Of note, a large share of providers reported not seeing or treating TB patients: 24.6% of those with medical qualifications and 67.2% of those without. While it is possible to analyse provider TB vignette performance only for those reporting seeing and treating TB patients, we chose instead to analyse all providers based on the following rationale. Since TB is endemic in rural Bihar, patients may present with symptoms of their illness even to providers who do not claim to treat TB since patients will not know that they are suffering from TB when they visit the provider. In such a situation, if the provider does not recognise the symptoms, and ask the right diagnostic questions or perform the minimum necessary examinations, TB patients would still receive delayed TB diagnosis and poor quality healthcare. We include providers with and without formal qualifications. Practitioners with little to no formal training provide most healthcare in rural areas in India, and previous studies on quality of care in rural India report that the quality of care provided by practitioners with formal qualifications is also poor .^ii^ 7 18–21 Hence, our analysis of provider performance on vignettes includes all providers in our sample. We also include analogous analyses and findings after restricting to providers who claim to treat TB in the online supplementary appendix.

For diagnosis and treatment, we concentrated our analyses on three aspects: (1) diagnostic process; (2) providing a correct diagnosis and (3) whether or not the correct treatment was prescribed. We assessed the diagnostic process each provider stated that he/she would undertake (ie, the questions, examinations and tests used to form a diagnosis) relative to standard diagnostic procedures to measure ‘knowledge’. We summarised provider knowledge using Item Response Theory (IRT) to calculate a knowledge score for each provider using previously developed methods.^17^ The IRT methodology is a model-based measurement used to describe the relation between how the provider responds to a set of questions and the level of the ‘latent variable’ (knowledge) being measured by the scale. It is widely used in settings to assess items in questionnaires where participants are scored on multiple items to recover an underlying latent trait or ability. In the context of our paper, a correct response to an item is obtained every time a provider asks a key diagnostic question (such as duration of cough), or for results of diagnostic tests (such as sputum smear) or performs a relevant examination (listed in table 2), and our latent variable or trait is the provider's overall knowledge. We use a three-parametric logistic (3PL) model to construct our knowledge index following Das and Hammer.^17^ Regardless of diagnostic process, we assessed whether each provider correctly stated a diagnosis of the case in the vignette as being TB.

In identifying the appropriate TB treatment, we followed the WHO 2010 guidelines and WHO's 2014 Standards for TB Care in India.^22^ The WHO treatment guidelines state that the treatment of new TB cases include 6 months of rifampicin as part of a multidrug regimen (2HRZE+4 HR).^iii^ However, since our investigators were unable to collect information on duration of each of the prescribed drugs consistently, with some providers using generic pharmaceutical names of drugs and others using brand names, we employ a broader definition of ‘correct’ treatment. We defined correct treatment to include all prescriptions that included 6 months of rifampicin as part of a multidrug treatment that also included any duration of isoniazid.

We examined how a provider's observable characteristics are associated with their knowledge using multivariable linear regression models because these characteristics are the features that are available to patients to choose between providers. We also examined how knowledge along with a provider's observable characteristics related to the probability of making a correct diagnosis, providing correct treatment and making a referral using multivariable probit regression models.^iv^ Further, we conducted analyses where we restricted the assessment of correct treatment provision only to those providers who offered any treatment.

All regression models controlled for the age of the provider, years of experience and medical qualification. Additionally, in separate specifications, we controlled for the type of medicine practiced, the number of working hours per week, average patient caseload per day, whether providers engaged in public events like running medical camps, whether the clinic was public or private, whether provider claimed to treat TB, sold medicines at the clinic, infrastructure index and average fee charged by provider. All analyses adjusted SEs for survey design by clustering at the level of a cluster in our study.^v^

## Results

### Provider characteristics

**Figure 1:**
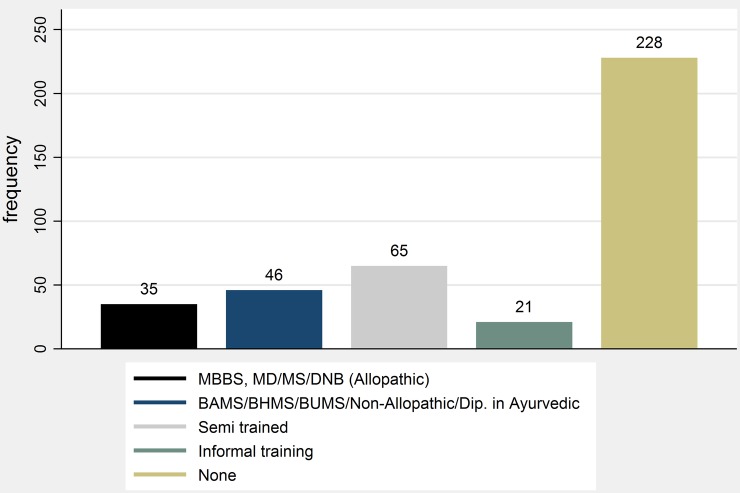
Distribution of providers by qualification.

Of the 395 providers most commonly visited by representative households in our study areas and interviewed in our study, 79.5% (314) did not have any formal medical qualifications. Among those with medical qualifications, less than half (35) had MBBS (Bachelor of Medicine, Bachelor of Surgery—the equivalent of MD in the USA) degrees or higher, while the remaining 46 had degrees or diplomas in Ayurveda, Homeopathy or Unani systems of medicine (BAMS/BHMS/BUMS),^23^ hereafter abbreviated as BA/H/UMS. Among the 314 providers without formal qualifications, 20.7% (65 out of 314) had some training such as pharmacist or registered medical practitioner (RMP); a small fraction reported informal training^vi^ where they had worked with other doctors in the past, while the vast majority (228) had no formal or informal medical qualification, as seen in figure 1.

Table 1 describes provider characteristics, among those with and without medical qualifications. While providers with formal qualifications have comparable years of experience as those without qualifications as well as comparable levels of ownership of the facilities that they practice in, they vary significantly in the range of services they provide. MBBS providers report longer working hours per week, higher participation in camps and significantly lower rates of providing treatment as part of their consultation and selling drugs than providers with training in other systems and those without training. In rural settings such as the one where this study was conducted, providers frequently carry their own stock of medicines and offer medication (treatment) as part of the consultation and charge a combined fee. We recorded this characteristic of the provider/facility as ‘administering treatment’ in the survey (90.8% among the unqualified and 76.1% among the BA/H/UMS group compared with 57.1% among MBBS providers). Some providers sell drugs separately as well (53.2% among the unqualified and 41.3% among the BA/H/UMS group compared with 8.6% among MBBS providers). Providers with MBBS training work in clinics with higher levels of infrastructure and also command fees that are almost twice as high as BA/H/UMS providers and over three times as high as those without formal medical qualifications.

**Table 1:** Providers characteristics according to medical education

Variables	[1] MBBS	[2] BA/H/UMS	[3] Other	[4] DIFF 1–2	[5] DIFF 1–3
Age (years)	45.7 (42.2 to 49.2)	46.8 (43.6 to 50)	43.5 (42.2 to 44.7)	−1.1	2.2
Education >high School (%)	100 (– to –)	97.8 (93.6 to 102.1)	70.7 (65.7 to 75.7)	2.2	29.30***
Has ever used a computer (%)	62.9 (46.6 to 79.1)	30.4 (17 to 43.9)	12.4 (8.8 to 16.1)	32.42***	50.44***
Experience (years)	18.5 (15 to 22.1)	19 (16 to 21.9)	18.2 (17.1 to 19.4)	−0.4	0.3
Average patient caseload (day)	29.3 (23.5 to 35.1)	17.1 (15.6 to 18.6)	17.2 (16.5 to 18)	12.17***	12.06***
Working hours (per week)	61.8 (56.3 to 67.4)	52.2 (47.2 to 57.2)	48.3 (46.4 to 50.3)	9.61**	13.51***
Run camps (%)	40 (23.5 to 56.5)	6.5 (−0.7 to 13.7)	4.5 (2.2 to 6.7)	33.48***	35.54***
Public health facility (%)	22.9 (8.7 to 37)	2.2 (−2.1 to 6.4)	0.3 (−0.3 to 0.9)	20.68***	22.54***
Infrastructure Index	3.1 (2 to 4.3)	0.2 (−0.3 to 0.7)	−0.4 (−0.5 to −0.3)	2.92***	3.53***
Consultation fee (Rs)	64.2 (44.8 to 83.6)	38.6 (26.4 to 50.9)	15.5 (13.3 to 17.6)	25.60**	48.75***
Task (% of providers)					
Consultation with patients	100 (– to –)	100 (– to –)	99.7 (99.1 to 100.3)	0.0	0.3
Administering treatment	57.1 (40.5 to 73.8)	76.1 (63.6 to 88.5)	90.8 (87.6 to 94)	–	–33.62***
Selling drugs	8.6 (−0.8 to 18)	41.3 (26.9 to 55.7)	53.2 (47.7 to 58.7)	–32.73***	–44.61***
Laboratory-related duties	17.1 (4.5 to 29.8)	6.5 (−0.7 to 13.7)	3.8 (1.7 to 5.9)	10.6	13.3
Administrative duties	48.6 (31.8 to 65.4)	71.7 (58.6 to 84.9)	61.8 (56.4 to 67.2)	–23.17**	−13.2
Ownership	51.4 (34.6 to 68.2)	76.1 (63.6 to 88.5)	72 (67 to 77)	–24.66**	–20.55**
Type of medicine practiced (%)					
Allopathic	94.3 (86.5 to 100)	89.1 (80 to 98.2)	91.4 (88.3 to 94.5)	5.2	2.9
Homeopathic/Ayurvedic	20 (6.6 to 33.4)	63 (48.9 to 77.1)	32.8 (27.6 to 38)	–43.04***	−12.8
Type of diseases treated (%)					
Tuberculosis	88.6 (77.9 to 99.3)	65.2 (51.3 to 79.1)	32.8 (27.6 to 38)	23.35***	55.77***
VL	2.9 (−2.7 to 8.5)	4.3 (−1.6 to 10.3)	1.9 (0.4 to 3.4)	−1.5	1.0
Observations	35	46	314		

Columns 1–3 report mean (95% CI) and columns 4 and 5 report differences. BA/H/UMS includes BAMS, BUMS and BHMS degrees as well as Diploma in Ayurvedic and some others MD degrees. Providers classified as ‘Other’ includes all providers with NO medical training or those with coursework related in some way to medicine such as pharmacist or informal training. The infrastructure index was computed according to the following variables: electricity, power backup, number of consulting rooms, number of bed for day observation, provision of tests, provision of X-rays and computer system.

Asterisks represent statistically significant differences, with ***p<0.01, **p<0.05, *p<0.1. Source: Providers Interview.

When asked whether they provide treatment for TB, 88.6% of MBBS providers reported treating patients, as did 65.2% of BA/H/UMS providers and 32.8% of those without qualifications. While it is tempting to conclude that most providers in the sample—especially those without medical qualifications—do not manage TB, such a conclusion would miss a critical point that patients often do not know what their underlying illness is, at least in the early stages of the care-seeking pathway. Patients experience symptoms and seek care from providers whom they frequently visit.^11^ ^24–27^ The provider then has to diagnose the condition, and decide whether to treat the patient or refer them to a hospital. If providers incorrectly diagnose TB as another condition, they may incorrectly also report that they do not treat TB when surveyed.

### Provider knowledge: diagnostic questions, treatment and referral

Table 2 shows the overall fraction of providers and fraction by type of provider, who asked or performed key diagnostic questions and examinations, made the right diagnosis and provided the correct treatment. The most common question asked by providers was duration of fever (48.9%, 95% CI 43.8% to 53.9%). Only 32.2% (95% CI 27.6% to 37.0%) of providers asked a key question related to TB diagnosis about duration of cough (30.9% (95% CI 24.9% to 36.9%) among unqualified, relative to 37.1% (95% CI 22.7% to 51.5%) among MBBS providers).

**Table 2:** Fraction of providers who asked or performed key diagnostic questions and examinations, diagnosis and treatment and average competence score

	All providers	MBBS [1]	BA/H/U/MS [2]	Other [3]	DIFF 1–2	DIFF 1–3
Questions and examinations						
Since when has he had the fever	48.9 (43.8 to 53.9)	51.4 (32.7 to 70.1)	47.8 (33.1 to 62.5)	48.7 (42.7 to 54.7)	3.6	2.7
Fever with chills	31.4 (26.8 to 36.2)	31.4 (16 to 46.9)	32.6 (18.6 to 46.6)	31.2 (25.5 to 37.0)	−1.2	0.2
Is fever continuous	33.4 (28.8 to 38.3)	34.3 (16.5 to 52.1)	39.1 (23.5 to 54.8)	32.5 (26.8 to 38.2)	−4.8	1.8
Are there any night sweats present	6.1 (3.9 to 8.9)	11.4 (2.2 to 20.6)	6.5 (−0.5 to 13.6)	5.4 (2.9 to 7.9)	4.9	6.0
Cough since when	32.2 (27.6 to 37)	37.1 (22.7 to 51.5)	37.0 (20 to 53.9)	30.9 (24.9 to 36.9)	0.2	6.3
Pain in the chest	13.7 (10.4 to 17.5)	20.0 (5.1 to 34.9)	21.7 (9.5 to 34)	11.8 (8.2 to 15.4)	−1.7	8.2
Is there sputum	24.6 (20.4 to 29.1)	34.3 (20.2 to 48.4)	23.9 (10.4 to 37.4)	23.6 (19.0 to 28.2)	10.4	10.7
How is the sputum	24.6 (20.4 to 29.1)	37.1 (20 to 54.3)	23.9 (9.7 to 38.1)	23.2 (18.8 to 27.7)	13.2	13.9
Blood in the sputum	11.1 (8.2 to 14.7)	14.3 (0.7 to 27.9)	13.0 (3.3 to 22.8)	10.5 (7.3 to 13.7)	1.2	3.8
How much blood in the sputum	10.9 (8.0 to 14.4)	8.6 (−0.4 to 17.6)	10.9 (1.4 to 20.3)	11.1 (7.4 to 14.8)	−2.3	−2.6
Have you been eating less	13.2 (10 to 16.9)	28.6 (12.3 to 44.8)	23.9 (10.8 to 37.1)	9.9 (6.3 to 13.4)	4.7	18.7**
Have you visited other doctors before coming here	15.9 (12.5 to 19.9)	20.0 (4.2 to 35.8)	8.7 (0.2 to 17.2)	16.6 (11.7 to 21.4)	11.3	3.4
Weight	10.6 (7.8 to 14.1)	25.7 (8.5 to 43)	13.0 (2.8 to 23.3)	8.6 (5.2 to 12.0)	12.7	17.1*
Temperature	16.7 (13.2 to 20.8)	20.0 (7.1 to 32.9)	13.0 (3.0 to 23.1)	16.9 (11.8 to 22.0)	7.0	3.1
Blood for tlc/dlc	32.4 (27.8 to 37.3)	45.7 (27.3 to 64.1)	28.3 (14.8 to 41.7)	31.5 (25.2 to 37.8)	17.5	14.2
Blood test hb	15.9 (12.5 to 19.9)	34.3 (18.5 to 50)	10.9 (1.2 to 20.5)	14.6 (10.0 to 19.3)	23.4**	19.6**
Blood for fasting ESR (erythrocytic sedimentation rate)	32.4 (27.8 to 37.3)	37.1 (20.3 to 54)	26.1 (12.6 to 39.6)	32.8 (26.1 to 39.5)	11.1	4.3
Mantaux tuberculin skin test	12.7 (9.5 to 16.3)	17.1 (3.5 to 30.8)	13.0 (2.8 to 23.3)	12.1 (8.4 to 15.8)	4.1	5.0
Sputum for AFB (acid-fast bacilli)	20.0 (16.2 to 24.3)	54.3 (35.6 to 73)	15.2 (4.5 to 26.0)	16.9 (12.4 to 21.3)	39.1***	37.4***
Chest X-ray	31.9 (27.3 to 36.7)	57.1 (38.2 to 76.1)	28.3 (13.6 to 42.9)	29.6 (23.9 to 35.3)	28.9**	27.5***
TB test	2.3 (1.0 to 4.3)	2.9 (−2.8 to 8.5)	4.3 (−1.7 to 10.4)	1.9 (0.4 to 3.4)	−1.5	1.0
Diagnosis						
Gave any diagnosis	92.4 (89.3 to 94.8)	97.1 (91.3 to 103)	95.7 (89.4 to 101.9)	91.4 (88.3 to 94.5)	1.5	5.7*
Correct diagnosis	60.0 (55.0 to 64.9)	91.4 (81.8 to 101)	73.9 (60.8 to 87)	54.5 (48.8 to 60.1)	17.5**	37.0***
Correct diagnosis, if any	64.9 (59.8 to 69.8)	94.1 (86.1 to 102.2)	77.3 (64.4 to 90.2)	59.6 (53.6 to 65.6)	16.8**	34.5***
Treatment						
Gave any treatment	66.6 (61.7 to 71.2)	82.9 (71.0 to 94.8)	80.4 (68.1 to 92.8)	62.7 (57.2 to 68.3)	2.4	20.1***
Correct treatment	14.4 (11.1 to 18.3)	45.7 (26.0 to 65.4)	19.6 (8.6 to 30.6)	10.2 (6.8 to 13.6)	26.2**	35.5***
Correct treatment, if any	21.7 (16.8 to 27.1)	55.2 (33.4 to 76.9)	24.3 (11.2 to 37.5)	16.2 (11.1 to 21.4)	30.9**	38.9***
Others						
Recommend referral	48.9 (43.8 to 53.9)	77.1 (63.3 to 91.0)	63.0 (48.8 to 77.3)	43.6 (37.4 to 49.9)	14.1	33.5***
Knowledge score	−1.0 (−1.2 to −0.73)	0.0 (−0.6 to 0.6)	−1.3 (−2.1 to −0.45)	−1.0 (−1.3 to −0.7)	1.3**	1.0***

Values are percentage except for the competence score variable. Source: Vignette survey. Observations from 395 providers. CIs reported in parentheses.

Asterisks represent statistically significant differences, with ***p<0.01, **p<0.05, *p<0.1.

Few providers asked for results of common diagnostic tests, though MBBS doctors were significantly more likely to ask than other types of providers (table 2). Overall, 31.9% of providers asked for a chest X-ray, 20% asked for results from a Sputum test and 12.7% asked for a Mantoux tuberculin skin test. (See vignette in online supplementary appendix for positive results reported for each of the tests.) While 57.1% of MBBS providers asked for a chest X-ray, only 29.6% of unqualified providers and 28.3% of BA/H/UMS providers did so. Similarly, 16.9% of unqualified providers and 15.2% of BA/H/UMS providers asked for sputum test results relative to 54.3% of MBBS providers. The average number of questions asked per provider was 2.66 and the average number of examinations performed was 1.75, with no significant differences across provider types.

Although almost all providers (92.4%) reported a diagnosis on the vignette interview, unqualified providers and providers without MBBS degrees were more likely to provide an incorrect diagnosis. Among the providers who gave a diagnosis, 94.1% of MBBS providers gave a correct diagnosis, compared with 77.3% of BA/H/UMS providers and 59.6% of unqualified providers. Two-thirds of all providers (66.6%) prescribed treatment on the vignette interviews, but only 14.4% of them prescribed correct treatment. Among the ones who prescribed any treatment, 55.2% of MBBS providers gave the correct TB treatment. In comparison, only 24.3% of BA/H/UMS providers and 16.2% of the unqualified providers prescribed the correct treatment. When we restricted the sample to providers who claimed to treat TB in their practice, the share of providers who gave the correct treatment was 19.5%, compared with 14.4% among all providers. Almost all prescriptions recommended also included additional medicines such as multivitamin syrups, cough medicines and antipyretics. Further, a substantial proportion of providers recommended referring patients to larger facilities for treatment (48.9%), with MBBS providers being statistically more likely to refer relative to other types of providers.

The majority of providers (58.6%) prescribed other drugs that did not include any medicines that are part of the WHO multidrug treatment regimen for new TB patients (see online supplementary appendix table A-1). Further, only 31.9% of providers prescribed any combination of isoniazid (H) and rifampicin (R) and only 21.7% prescribed it for 6 months (180 days) or more (per the WHO guidelines).

### Knowledge scores and provider characteristics

**Figure 2:**
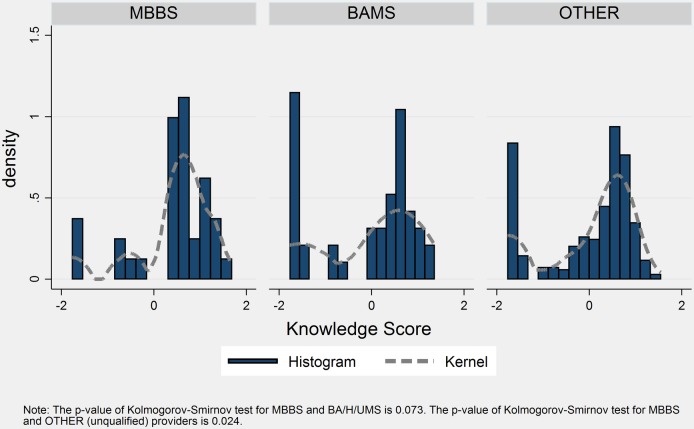
Knowledge distribution by qualification.

**Figure 3:**
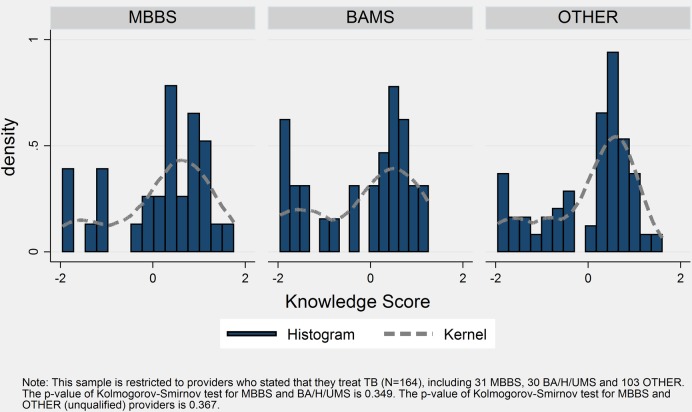
Knowledge distribution by qualification, among providers who report treating tuberculosis.

Summarising the knowledge reflected in diagnostic workup, diagnosis and treatment using IRT scores, MBBS doctors showed significantly higher levels of TB diagnostic and treatment knowledge than other provider types (table 2 and figure 2) based on the Kolmogorov-Smirnov (KS) tests of equality of distributions (p values of 0.07 and 0.02 compared with BA/H/UMS and unqualified providers, respectively). However, there is no statistically significant difference in the distribution of knowledge scores when restricted to the sample of providers who claim to treat TB (figure 3). The KS test of equality of distributions is not significant when comparing providers who claim to treat TB (p value of 0.34 and 0.36, respectively). The KS tests do not, however, account for clustering, which would typically result in higher p values by making estimates less precise.

We are interested in whether patients might be able to choose providers with higher levels of TB diagnostic and treatment knowledge by observing certain provider characteristics. Both in the parsimonious model in column 1 of table 3 as well as in column 2, which controls for additional observable characteristics, age and experience are not associated with knowledge scores. Overall, the observable characteristics only explain 15.9% of the variation in knowledge measured on the vignettes, suggesting that patients would have a very difficult time assessing the knowledge of providers by using observable characteristics. Relative to providers with an MBBS degree, those with BA/H/UMS qualifications as well those with other (including non-medically trained) qualifications have knowledge scores that are half an SD lower (−0.55 for BA/H/UMS and −0.46 for other). Hence, patients could in principle select to go to an MBBS provider should one be available in their area if they desired higher knowledge levels. However, as seen in column 2, controlling for type of medical qualifications, practicing Homeopathic/Ayurvedic type of medicine is associated with over a third of an SD (0.356) higher knowledge score. In column 3, after controlling for additional characteristics, including whether the provider reported treating TB, medical qualification is no longer associated with knowledge score. Controlling for medical qualification and self-reported TB treatment and all other characteristics, practicing Ayurveda/Homoeopathy/Unani continues to be significantly associated with a 0.29 SD higher knowledge score and an increase of about 10 working hours/week is associated with a 0.07 SD increase in the knowledge score. Column 3 also indicates that the knowledge score among providers who claim to treat TB is about 0.46 SDs higher compared with those who did not report this.

**Table 3:** Knowledge score and providers characteristics

Variables	Estimated effect (95% CI)		
	[1]	[2]	[3]
Age	0.03 (−0.04 to 0.09)	0.02 (−0.04 to 0.09)	0.02 (−0.05 to 0.08)
Age^2^	−0.00 (−0.00 to 0.00)	−0.00 (−0.00 to 0.00)	−0.00 (−0.00 to 0.00)
Experience (years)	0.00 (−0.02 to 0.02)	−0.01 (−0.03 to 0.02)	−0.01 (−0.03 to 0.01)
Medical qualification: BA/H/UMS	−0.55 (−1.00 to −0.10)	−0.59 (−1.08 to −0.10)	−0.20 (−0.66 to 0.25)
Medical qualification: other	−0.46 (−0.75 to −0.17)	−0.39 (−0.77 to −0.00)	0.27 (−0.09 to 0.62)
Practice allopathy		−0.01 (−0.35 to 0.33)	−0.09 (−0.42 to 0.24)
Practice Ayur/Homoeo/Unani		0.36 (0.13 to 0.58)	0.29 (0.07 to 0.51)
Working hours (/week)		0.01 (−0.00 to 0.01)	0.01 (0.00 to 0.01)
Average patient caseload (day)		0.00 (−0.01 to 0.01)	−0.00 (−0.01 to 0.01)
Run camps		0.14 (−0.24 to 0.52)	−0.03 (−0.41 to 0.35)
Public health facility		0.35 (−0.13 to 0.83)	0.28 (−0.36 to 0.92)
Treat TB			0.46 (0.28 to 0.63)
Sell drug			−0.20 (−0.40 to 0.01)
Infrastructure index			0.08 (0.01 to 0.15)
Consultation fee (Rs)			0.00 (−0.00 to 0.01)
Observations	395	395	395
R^2^	0.035	0.072	0.159

Columns 1–3 show OLS regression estimates of association between knowledge score and providers characteristics. The reference group for Medical Qualification is MBBS. The first column includes variables related mainly with sociodemographic characteristics of the providers. Column 2 includes variables related to effort/case load and type of facility. The last column includes variables related with the provider's capability (self-reported ability to treat TB), whether they sell drugs, reported fees and infrastructure index. The infrastructure index was computed according to the following variables: electricity, power backup, number of consulting rooms, number of bed for day observation, provision of tests, provision of X-rays and computer system. SEs in parentheses, clustered at the level of study cluster. Sources: Vignette survey and Provider Questionnaire.

The regression results in online supplementary appendix table A-2 show the analogous analyses from table 3 restricted to the sample of providers who claimed to treat TB. The results indicate that none of the observable characteristics (such as medical qualification, type of medicine practiced, average patient caseload and infrastructure index) are significantly associated with the knowledge score in this sample. These results indicate that patients are likely to find it especially difficult to discern the knowledge of providers among those who treat TB, and the dominant marker of provider knowledge is whether they claim to treat TB or not. Unfortunately, this marker might not be readily observable to patients, especially ones who might not know what their illness is.

The results reported in table 4, where we conduct OLS regressions of consultation fees on the set of observable characteristics, further suggest that patients might not be able to assess providers' knowledge based on observed prices in the market. In the parsimonious model in column 1 of table 4, the coefficient on medical qualification indicates that qualified providers charge on average INR 24.1 more than BA/H/UMS providers and INR 46.9 more than unqualified providers. Also a 1 SD increase in the knowledge score, controlling for qualifications, age and experience, is associated with a higher consultation fee of INR 3.4. Even after controlling for additional observable characteristics in columns 2 and 3, the coefficients on medical qualification and the knowledge score remain significant and within a similar range.

**Table 4:** Consultation fee and providers characteristics

Variables	Estimated effect (95% CI)		
	[1]	[2]	[3]
Age	−0.73 (−2.93 to 1.47)	−0.79 (−2.94 to 1.36)	−0.83 (−2.91 to 1.26)
Age^2^	0.01 (−0.01 to 0.03)	0.01 (−0.01 to 0.03)	0.01 (−0.01 to 0.03)
Experience (years)	0.21 (−0.30 to 0.72)	0.14 (−0.28 to 0.56)	0.11 (−0.31 to 0.53)
Medical qualification: BA/H/U/MS	−24.07 (−45.10 to −3.04)	−34.00 (−53.00 to −15.00)	−28.12 (−48.40 to −7.84)
Medical qualification: other	−46.95 (−68.49 to −25.42)	−58.38 (−78.42 to −38.33)	−47.97 (−69.85 to −26.08)
Knowledge score	3.40 (1.42 to 5.39)	4.06 (1.97 to 6.15)	2.28 (−0.09 to 4.65)
Practice allopathy		14.71 (5.54 to 23.87)	12.97 (3.24 to 22.69)
Practice Ayur/Homoeo/Unani		0.33 (−6.33 to 6.99)	−0.15 (−6.36 to 6.06)
Working hours (/week)		−0.09 (−0.26 to 0.08)	−0.07 (−0.24 to 0.10)
Average patient caseload (day)		0.35 (−0.08 to 0.77)	0.25 (−0.18 to 0.68)
Run camps		2.69 (−14.50 to 19.88)	−0.97 (−18.65 to 16.72)
Public health facility		−77.43 (−105.07 to −49.79)	−87.04 (−113.93 to −60.15)
Treat TB			7.57 (0.98 to 14.16)
Sell drug			−7.18 (−12.85 to −1.51)
Infrastructure index			2.42 (−0.68 to 5.52)
Observations	395	395	395
R^2^	0.245	0.363	0.396

Columns 1–3 show OLS regression estimates of association between consultation fee and providers characteristics. The reference group for Medical Qualification is MBBS. The first column includes variables related mainly with sociodemographic characteristics of the providers. Column 2 includes variables related with effort/case load and type of facility. The last column includes variables related with the provider's capability (self-reported ability to treat TB), whether they sell drugs, reported fees and infrastructure index. The infrastructure index was computed according to the following variables: electricity, power backup, number of consulting rooms, number of bed for day observation, provision of tests, provision of X-rays and computer system. SEs in parentheses, clustered at the level of study cluster. Sources: Vignette survey and Provider Questionnaire.

After controlling for the full set of observable characteristics, including treating TB and provider knowledge score, MBBS providers charge an average of INR 28.1 more than BA/H/UMS providers and INR 47.9 more than unqualified providers. This suggests a large premium charged by MBBS providers given that, as we saw in table 2, there is no statistically significant difference in the knowledge score of qualified versus non-qualified providers once we control for treating TB. The regression-adjusted difference in the knowledge score between MBBS and BA/H/UMS providers is 0.202 (from table 3), which would only predict an increase of INR 0.46 (0.202×2.282=0.76) instead of INR 28.1. If we do not control for TB treatment, the predicted fee would be only INR 2.40 higher (0.591×4.061). In a framework where providers with higher knowledge could charge higher fees, one possible explanation for our finding that knowledge is not adequately priced could be due to substantial asymmetric information: patients cannot directly assess providers' knowledge and cannot infer it from outcomes (either theirs or of other patients) due to variations in case-mix and infrequent experience. However, another explanation might be that knowledge might be only very loosely related to performance,^13^ which is what ultimately matters for the patient.

Additionally, the regressions in column 3 of table 4 indicate that the practice of allopathic medicine is associated with INR 12.97 higher consultation fee even after controlling for qualifications. This probably explains why so many BA/H/UMS providers end up adopting allopathic therapeutic methods. Public health facilities though, charge about INR 87.04 lower. Providers who also sell medicines as part of their practice charge INR 7.18 lower than those who do not sell drugs on their premises, presumably because profits from drug sales could offset the fees.

In online supplementary appendix table A-3, which shows the results from the same regressions as in table 4 but run using a sample restricted to providers who treat TB, the MBBS qualification is still significantly associated with higher consultation fee. MBBS providers are likely to charge INR 41.33 more than providers who are not qualified, and INR 14.65 more than BA/H/UMS providers (although the latter is not statistically significant), suggesting a substantial premium for the MBBS qualification.

### Correct diagnosis and treatment

Next, we turn to the relationship between provider characteristics and making a correct diagnosis. As columns 1 and 2 of table 5 show, unqualified providers are significantly less likely to make a correct diagnosis relative to MBBS providers (∼40%), and BA/H/UMS providers do not show statistically significant differences relative to MBBS providers. The knowledge score is significantly and positively associated with making the correct diagnosis. A 1 SD increase in the knowledge score is associated with a 13 percentage point increase (columns 1 and 2) in the probability of making a correct diagnosis. The significant effects of medical qualification and knowledge scores persist even after controlling for other observable characteristics as seen in column 3 of table 5.

**Table 5:** Correct diagnostic (if any)—marginal effects

Variables	Estimated effect (95% CI)		
	[1]	[2]	[3]
Age	0.01 (−0.02 to 0.04)	0.01 (−0.01 to 0.04)	0.01 (−0.01 to 0.04)
Age^2^	−0.00 (−0.00 to 0.00)	−0.00 (−0.00 to 0.00)	−0.00 (−0.00 to 0.00)
Experience (years)	0.00 (−0.01 to 0.01)	0.00 (−0.01 to 0.01)	0.00 (−0.01 to 0.01)
Medical qualification: BA/H/UMS	−0.19 (−0.46 to 0.07)	−0.22 (−0.50 to 0.07)	−0.12 (−0.39 to 0.15)
Medical qualification: other	−0.40 (−0.63 to −0.17)	−0.43 (−0.68 to −0.17)	−0.27 (−0.53 to −0.00)
Knowledge score	0.13 (0.09 to 0.17)	0.13 (0.09 to 0.18)	0.11 (0.07 to 0.16)
Practice allopathy		0.08 (−0.09 to 0.25)	0.05 (−0.12 to 0.22)
Practice Ayurv/Homoeo/Unani		−0.01 (−0.12 to 0.11)	−0.02 (−0.13 to 0.10)
Working hours (/week)		−0.00 (−0.00 to 0.00)	−0.00 (−0.00 to 0.00)
Average patient caseload (day)		−0.00 (−0.01 to 0.00)	−0.00 (−0.01 to 0.00)
Run camps		−0.07 (−0.24 to 0.11)	−0.11 (−0.28 to 0.07)
Public health facility		0.10 (−0.30 to 0.50)	0.07 (−0.40 to 0.55)
Treat TB			0.08 (−0.02 to 0.18)
Sell drug			−0.07 (−0.16 to 0.02)
Infrastructure index			0.02 (−0.02 to 0.07)
Consultation fee (Rs)			0.00 (−0.00 to 0.00)
Observations	365	365	365

Columns 1–3 show marginal effects of probit regression estimates of association between correct diagnosis variable for tuberculosis case and providers characteristics. The reference group for medical qualification is MBBS. The first column includes variables related mainly with sociodemographic characteristics of the providers. Column 2 includes variables related with effort/case load and type of facility. The last column includes variables related with the provider's capability (self-reported ability to treat TB), whether they sell drugs, reported fees and infrastructure index. The infrastructure index was computed according to the following variables: electricity, power backup, number of consulting rooms, number of bed for day observation, provision of tests, provision of X-rays and computer system. SEs in parentheses, clustered at the level of study cluster. Sources: Vignette Survey and Provider Questionnaire.

We see similar results in online supplementary appendix table A-4 when the regression is run on the restricted sample of providers who stated that they treat TB. Again, MBBS qualifications (relative to unqualified providers) and knowledge scores are significantly positively associated with the probability of making a correct diagnosis. In this restricted sample, we also see that the probability of making a correct diagnosis is lower by 16.1 percentage points if a provider also sells drugs.

The parsimonious models in columns 1 and 2 in table 6 show that providers with BA/H/UMS or no qualifications are 20–30 percentage points less likely to prescribe correct treatment relative to MBBS providers.

**Table 6:** Correct treatment (if any)—marginal effects

	Estimated effect (95% CI)		
Variables	[1]	[2]	[3]
Age	−0.00 (−0.04 to 0.04)	−0.00 (−0.04 to 0.03)	−0.00 (−0.04 to 0.03)
Age^2^	−0.00 (−0.00 to 0.00)	0.00 (−0.00 to 0.00)	0.00 (−0.00 to 0.00)
Experience (years)	0.00 (−0.01 to 0.01)	0.00 (−0.01 to 0.01)	−0.00 (−0.01 to 0.01)
Medical qualification: BA/H/UMS	−0.20 (−0.39 to −0.02)	−0.20 (−0.41 to 0.00)	−0.14 (−0.34 to 0.07)
Medical qualification: other	−0.30 (−0.44 to −0.16)	−0.29 (−0.46 to −0.13)	−0.11 (−0.31 to 0.10)
Knowledge score	0.04 (−0.02 to 0.10)	0.04 (−0.02 to 0.11)	0.02 (−0.04 to 0.08)
Practice allopathy		0.23 (0.04 to 0.42)	0.20 (0.02 to 0.38)
Practice Ayurv/Homoeo/Unani		0.01 (−0.11 to 0.12)	0.00 (−0.11 to 0.11)
Working hours (/week)		−0.00 (−0.00 to 0.00)	−0.00 (−0.00 to 0.00)
Average patient caseload (day)		−0.00 (−0.01 to 0.00)	−0.00 (−0.01 to 0.00)
Run camps		0.08 (−0.08 to 0.23)	0.04 (−0.11 to 0.19)
Public health facility		−0.10 (−0.38 to 0.18)	0.01 (−0.38 to 0.40)
Treat TB			0.14 (0.03 to 0.24)
Sell drug			−0.03 (−0.12 to 0.07)
Infrastructure index			0.01 (−0.03 to 0.05)
Consultation fee (Rs)			0.00 (0.00 to 0.00)
Observations	263	263	263

Columns 1–3 show marginal effect of probit regression estimates of association between correct diagnosis variable for tuberculosis case and providers characteristics. The reference group for medical qualification is MBBS. The first column includes variables related mainly with sociodemographic characteristics of the providers. Column 2 includes variables related with effort/case load and type of facility. The last column includes variables related with the provider's capability (self-reported ability to treat TB), whether they sell drugs, reported fees and infrastructure index. The infrastructure index was computed according to the following variables: electricity, power backup, number of consulting rooms, number of bed for day observation, provision of tests, provision of X-rays and computer system. SEs in parentheses, clustered at the level of study cluster. Sources: Vignette Survey and Provider Questionnaire.

However, neither medical qualifications nor knowledge scores are associated with the prescription of correct treatment after controlling for the full set of characteristics in column 3. Being a provider who treats TB though is associated with a 13.6 percentage point higher likelihood of prescribing the correct treatment. We see similar results in online supplementary appendix table A-5 when the regression is run on the sample restricted to providers who state that they treat TB.

### Referral

The likelihood of making a referral is significantly associated with medical qualifications. Compared with those with MBBS degrees, unqualified and BA/H/UMS providers are less likely to refer the TB case even after controlling for all observable characteristics (column 3 of table 7). BA/H/UMS providers were 23.2 percentage points less likely, and unqualified were 37.7 percentage points less likely. While the knowledge score is significantly and positively associated with referrals in parsimonious models, it does not have a significant association after controlling for the full set of observable characteristics.

**Table 7:** Recommend referral—marginal effects

Variables	Estimated effect (95% CI)		
	[1]	[2]	[3]
Age	0.01 (−0.02 to 0.04)	0.02 (−0.02 to 0.05)	0.01 (−0.02 to 0.05)
Age^2^	−0.00 (−0.00 to 0.00)	−0.00 (−0.00 to 0.00)	−0.00 (−0.00 to 0.00)
Experience (years)	0.00 (−0.00 to 0.01)	0.00 (−0.00 to 0.01)	0.00 (−0.00 to 0.01)
Medical qualification: BA/H/UMS	−0.13 (−0.34 to 0.08)	−0.30 (−0.56 to −0.04)	−0.23 (−0.50 to 0.03)
Medical qualification: other	−0.32 (−0.48 to −0.16)	−0.50 (−0.71 to −0.28)	−0.38 (−0.63 to −0.12)
Knowledge score	0.04 (−0.00 to 0.09)	0.05 (−0.00 to 0.09)	0.03 (−0.02 to 0.08)
Practice allopathy		−0.06 (−0.25 to 0.14)	−0.07 (−0.27 to 0.13)
Practice Ayurv/Homoeo/Unani		−0.00 (−0.12 to 0.11)	−0.02 (−0.13 to 0.10)
Working hours (/week)		−0.00 (−0.00 to 0.00)	−0.00 (−0.00 to 0.00)
Average patient caseload (day)		−0.01 (−0.01 to −0.00)	−0.01 (−0.01 to −0.00)
Run camps		−0.11 (−0.31 to 0.08)	−0.15 (−0.34 to 0.03)
Public health facility		−0.15 (−0.57 to 0.26)	−0.27 (−0.74 to 0.20)
Treat TB			0.10 (−0.03 to 0.22)
Sell drug			−0.01 (−0.11 to 0.09)
Infrastructure index			0.03 (−0.01 to 0.07)
Consultation fee (Rs)			0.00 (−0.00 to 0.00)
Observations	395	395	395

Columns 1–3 show marginal effects of probit regression estimates of association between recommend referral variable for tuberculosis case and providers characteristics. The reference group for medical qualification is MBBS. The first column includes variables related mainly with sociodemographic characteristics of the providers. Column 2 includes variables related with effort/case load and type of facility. The last column includes variables related with the provider's capability (self-reported ability to treat TB), whether they sell drugs, reported fees and infrastructure index. The infrastructure index was computed according to the following variables: electricity, power backup, number of consulting rooms, number of bed for day observation, provision of tests, provision of X-rays and computer system. SEs in parentheses, clustered at the level of study cluster. Sources: Vignette Survey and Provider Questionnaire.

In online supplementary appendix table A-6, among providers who claim to treat TB, unqualified providers are significantly less (39.4 percentage points) likely to make a referral. Providers who sell drugs also have 16.6 percentage points lower probability of making a referral.

## Discussion

In rural Bihar, as in much of other parts of India, TB continues to be a major public health challenge. This study provides further evidence that provider knowledge of how to diagnose and treat a case of TB, as measured by clinical vignettes, is low. This is the case for providers who lack formal medical qualifications (who provide most of the care in rural areas^9^) as well as those providers who have formal medical qualifications. Providers ask few diagnostic questions (only 32% asked about duration of cough for TB, for example) and seldom seek diagnostic test information (only 20% asked for sputum test for TB). Provider performance on making a correct diagnosis is inadequate but not as low as provider performance on prescribing appropriate treatment. Over 60% of providers arrived at the correct diagnosis; but while more than 66% of providers prescribed a treatment, only 21.7% of those were correct according to TB treatment guidelines. Low rates of correct treatment have significant implications for problems of TB drug resistance.

The problem of poor TB diagnostic and treatment accuracy is unlikely to be solved by shifting patients towards MBBS providers in the area. While MBBS providers had higher average scores on diagnostic workup, diagnostic accuracy and prescribing correct treatment than other provider types, only 45.7% MBBS doctors still only prescribed correct treatment. This pattern of MBBS providers performing marginally better, but still at a level that is unacceptably low is consistent with evidence from recent studies in urban India using standardised patients (SPs), which provide information on actual practice.^27^ Second, after controlling for whether the provider offers treatment for TB, there are no statistically significant differences in terms of provider knowledge between MBBS and other types of difference. It is important to bear in mind that 82.9% of MBBS providers offer treatment for TB, relative to 80.4% of BA/H/UMS providers and 62.74% of providers with no formal training. However, since the informal sector providers are ubiquitous in the rural health landscape (almost 10 times as many as MBBS providers in our study sample), it is salient that after controlling for offering TB treatment, these providers are comparable in knowledge and correct treatment. A more disheartening view of this finding is that providers with formal medical degrees do not perform much better than those without formal training on vignette-based assessments of knowledge of diagnosing and treating TB. However, MBBS providers are able to command a premium in terms of consulting fees that is far higher than what a higher knowledge score might indicate.

We also find that prescription of incorrect treatments is related to the practice of selling drugs as part of their medical practice. Lower rates of selling drugs among those with formal qualifications reflect the fact that in the informal health sector, there is a missing market for consultations. Informal providers typically tend to charge a fee for ‘treatment’ that includes the drugs provided as opposed to a consultant service, which ends with diagnosis and prescription.

Our study faces limitations related to using data from vignette-based interviews. The main limitation of the vignette method is that it does not capture the actual level of quality of care provided. As documented in recent research on ‘know-do gaps’,^7^ ^28^ the quality of care provided to patients might in fact be considerably lower than what is reported and measured on vignettes. In fact, the know-do gap for TB care has been reported in India, using data from SPs.^28^ While some of the measures we report (such as rates of sputum test recommendations) are comparable with those reported from previous research with SPs,^28^ we note that measures such as correct diagnosis rates and appropriate referral are higher with data from vignettes. As a result, the (low) level of knowledge that we report represents the upper bound of what providers might actually provide. Further, our estimates of provider knowledge in rural Bihar are representative of similar socioeconomic and geographic areas in developing countries like India, but might not be widely generalisable.

Low levels of provider knowledge of TB diagnosis and treatment could not only hamstring ongoing efforts to control TB, but also make it worse by contributing to multidrug resistance.^29^ Recent experimental evidence on improvement on provider knowledge and adherence to protocol from intensive training programmes offered to informal sector providers in West Bengal provides a potential solution to addressing this challenge.^30^ Medical organisations in India have typically opposed proposals to offer training or improving capacity of informal sector providers, advocating instead for policies to increase MBBS trained providers. The challenge, however, is that trained MBBS/MD graduates are unlikely to choose to practice and live in rural areas. While the number of medical training institutions in India have increased dramatically in the past few decades,^23^ India's rural population continues to receive healthcare primarily from informal sector providers (Das *et al*. 2015. Forthcoming). Policymakers in India, and elsewhere, might want to prioritise strategies such as training, incentives, task shifting and regulation to improve knowledge and performance of existing providers in the healthcare system.

## Footnotes

i Using the average 2011 exchange rate of INR 49.12 to US$1.

ii Based on evidence that informal and formally qualified doctors provide low quality of care, we examine provider characteristics to understand if other factors such as experience, qualifications or volume are correlated with provider quality.

iii This recommended treatment for new TB cases (fourth edition published in 2010) has been in place at least since the publication of the third edition of the WHO tuberculosis treatment guidelines in 2003.

iv Evidence from recent studies from India and other developing countries shows that providers with qualifications and those without qualifications provide care that is of very low quality.^17^ Hence, our analysis of quality and provider characteristics helps understand if there are other observable provider characteristics such as age, experience and patient volume that might serve as useful signals of quality to patients.

v All analyses were conducted in STATA V.14.0.

vi We do not have information on whether informal providers have had any TB training, but since the TB programme in India is run primarily through the formal public sector, we do not expect that these providers would have received training.
